# Supplementary figures and images for: Composition Wheels: Visualizing dissolved organic matter using common composition metrics across a variety of Canadian ecozones
Source: PLoS One. 2021 Jul 9;16(7):e0253972. doi: 10.1371/journal.pone.0253972 (PMC8270205; doi:10.1371/journal.pone.0253972)

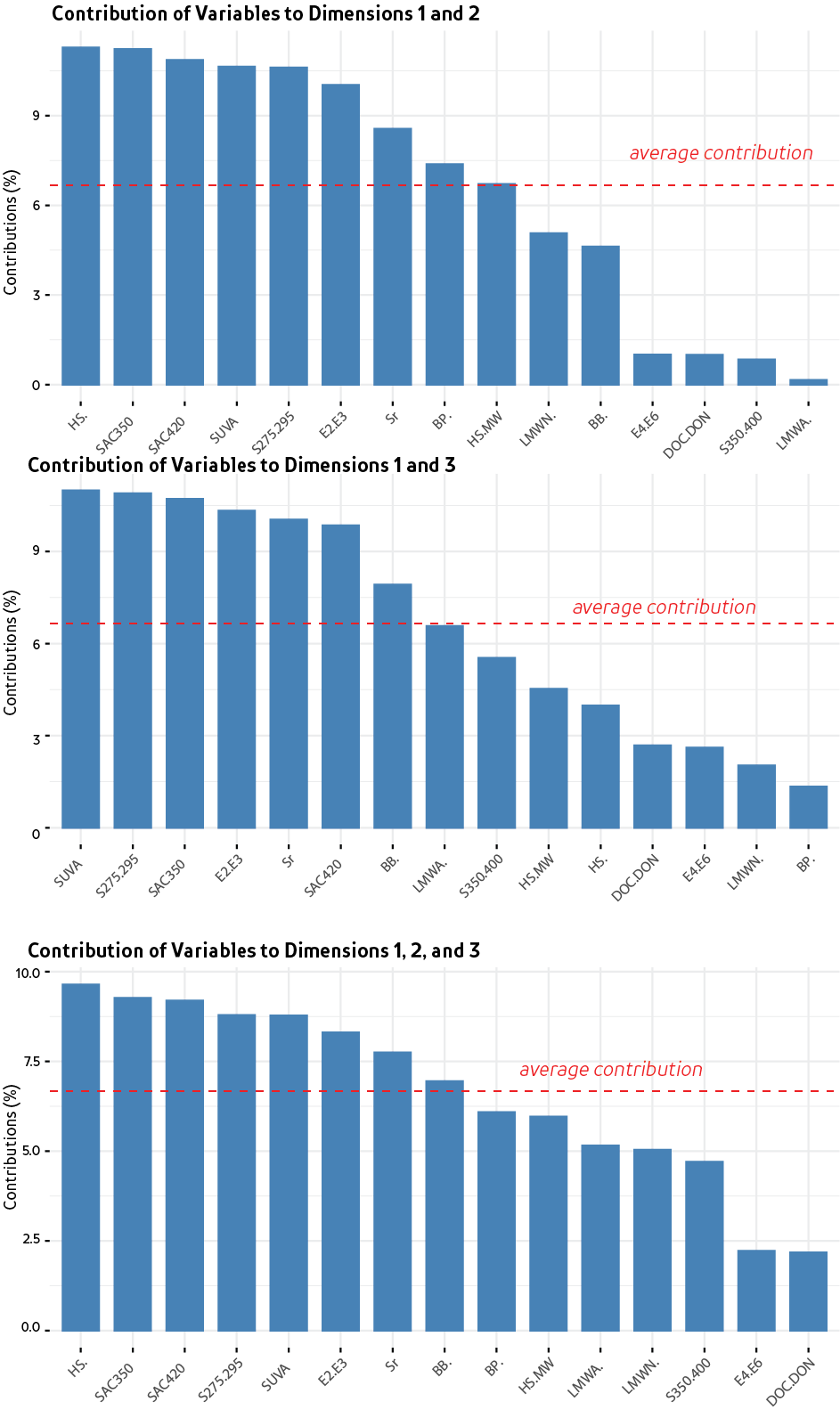

Supplement: S1 Fig — (PNG) [file pone.0253972.s001.png]
