# Supplementary material for: Composition Wheels: Visualizing dissolved organic matter using common composition metrics across a variety of Canadian ecozones
Source: PLoS One. 2021 Jul 9;16(7):e0253972. doi: 10.1371/journal.pone.0253972 (PMC8270205; doi:10.1371/journal.pone.0253972)
Supplement: S1 Table — Included are the number of total samples taken for DOM concentration with at least one compositional measure (nDOC), and number of samples used from that site in the PCA analysis (nPCA). (DOCX) [file pone.0253972.s002.docx]

**S1 Table. Environmental description for all sampling sites.** Included are the number of total samples taken for DOM concentration with at least one compositional measure (n_DOC_), and number of samples used from that site in the PCA analysis (n_PCA_).

| **SURFACE WATERS** | | | | |
| --- | --- | --- | --- | --- |
| **Name** | **Location** | **n_DOC_** | **n_PCA_** | **Description** |
| *IISD-Experimental Lakes Area, ON (ELA)* | 49° 39’ 40”N,  93° 43’ 48”W Ontario, Canada | **74** | **45** | Boreal forest, underlain by Precambrian bedrock with discontinuous surficial layer of sandy-gravel till. Sampled from 2010 to 2012. Ecozone mean annual temperature: 0.7 C; Ecozone total annual precipitation: 825 mm. |
| Lakes |  | *49* | *36* |  |
| Creeks |  | *25* | *9* |  |
| *Grand River, ON (GR)* | 43° 30’ 41”N,  80° 29’ 43”W Ontario, Canada | **39** | **-** | Surrounding land predominately agricultural and flows past six wastewater treatment plants. Sampled from 6 consecutive locations along a 90km stretch every two months from 2011 to 2012. Ecozone mean annual temperature: 6.5 C; Ecozone total annual precipitation: 940 mm. |
| *Yellowknife, NT (YK)* | 62° 27’ 14”N,  114° 22’ 18”W Northwest Territories, Canada | **23** | **20** | Samples from the Taiga Shield underlain by discontinuous permafrost. Surface waters are surrounded by bedrock and peat plateaux around Yellowknife. Sampled in July or October between 2013 and 2017. Ecozone mean annual temperature: -4.8 C; Ecozone total annual precipitation: 565 mm. |
| Lakes |  | *2* | *2* |  |
| Ponds |  | *8* | *7* |  |
| Rivers |  | *10* | *9* |  |
| Creek |  | *3* | *2* |  |
| *Mackenzie River, NT (MK)* | 63° 14’ 17”N,  123° 34’ 0”W Northwest Territories, Canada | **13** | **-** | Samples taken by the Community Based Monitoring network along the Mackenzie River in July and August of 2015. Samples ranged from WHERE to Inuvik. River flows through Taiga Shield and Taiga Plains. |
| Rivers |  | *13* | *-* |  |
| *Wekweètì, NT (WK)* | 64° 11’ 24”N,  114° 11’ 10”W Northwest Territories, Canada | **11** | **11** | Situated in the Taiga Shield, below treeline, continuous permafrost. Samples taken in October of 2015 and 2016. Ecozone mean annual temperature: -4.8 C; Ecozone total annual precipitation: 565 mm. |
| Lakes |  | *9* | *9* |  |
| Creeks |  | *2* | *2* |  |
| *Daring Lake, NT (DL)* | 64° 31’ 29”N,  111° 40’ 24”W Northwest Territories, Canada | **19** | **19** | Found in the Southern Arctic above treeline, continuous permafrost. Ecozone mean annual temperature: -9.8 C; Ecozone total annual precipitation: 313 mm. |
| Lakes |  | *7* | *7* |  |
| Ponds |  | *1* | *1* |  |
| Creeks |  | *11* | *11* |  |
| *Lake Hazen, NU (LH)* | 81° 49’ 30”N,  71° 19’ 26”W Nunavut, Canada | **160** | **12** | Tundra located in the high arctic; Lake Hazen Watershed is considered a local polar oasis. |
| Lakes |  | *38* | *2* |  |
| Ponds |  | *18* | *4* |  |
| Creeks |  | *41* | *1* |  |
| Rivers |  | *32* | *0* |  |
| Seeps |  | *31* | *5* |  |
| **GROUND WATERS** | | | | |
| **Name** | **Location** | **n** | **nPCA** | **Description** |
| *Turkey Lakes Watershed, ON (TLW)* | 47° 2’ 54”N, 84° 24’ 25”W Ontario, Canada | **16** | **-** | Relatively un-impacted watershed in the Great Lakes-St. Lawrence forest region. Area consists of Precambrian bedrock and surficial glacial deposits of glaciofluvial outwash. Samples collected from depths ranging between 0.90 - 6.89m below surface. Ecozone mean annual temperature: 0.7 C; Ecozone total annual precipitation: 825 mm. |
| *IISD-Experimental Lakes Area, ON (ELA)* | 49° 39’ 40”N, 93° 43’ 48”W Ontario, Canada | **17** | **-** | Piezometers constructed in transect along a wetland, ranging from 0.70 - 3.85m below surface. Ecozone mean annual temperature: 0.7 C; Ecozone total annual precipitation: 825 mm. |
| *Nottawasaga Aquifer, ON (NW)* | 44° 7’ 26”N,  79° 49’ 12”W Ontario, Canada | **6** | **-** | Surficial deposits of glaciolacustrine deposits in an agriculturally-impacted aquifer. Samples collected from single multi-level piezometer within an unconfined surficial sand aquifer at depths of 4.35m, 5.13m, 6.68m, 9.90m, and 11.3m below surface. Ecozone mean annual temperature: 6.5 C; Ecozone total annual precipitation: 940 mm. |
| *Black Brook Watershed, NB (BBK)* | 47° 6’ 11”N, 67° 45’ 40”W New Brunswick, Canada | **15** | **-** | Site is an agriculturally-impacted aquifer, sampled during the summer of 2012. Surficial deposits of till and small deposits of glacial outwash. Samples taken from twelve domestic wells and three multi-level piezometers (6.1 - 30m below surface). Ecozone mean annual temperature: 4.6 C; Ecozone total annual precipitation: 1185 mm. |
| *Long Point, ON (LP)* | 42° 34’ 46”N,  80° 22’ 57”W Ontario, Canada | **23** | **-** | Unconfined sand aquifer atop of a clay aquitard. Piezometers range from 1 to 4m below surface. Sampling of groundwater containing a septic plume. |
| *Yellowknife, NT (YK)* | 62° 27’ 14”N, 114° 22’ 18”W Northwest Territories, Canada | **33** | **16** | Samples from the Taiga Shield underlain by discontinuous permafrost. Surface waters are surrounded by bedrock and peat plateaux around Yellowknife. Sampled in July or October between 2013 and 2017. Ecozone total annual precipitation: 565 mm. |
| *Wekweètì, NT (WK)* | 64° 11’ 24”N,  114° 11’ 10”W Northwest Territories, Canada | **1** | **1** | Situated in the Taiga Shield, below treeline, continuous permafrost. Samples taken in October of 2015 and 2016. Ecozone total annual precipitation: 565 mm. |
| *Daring Lake, NT (DL)* | 64° 31’ 29”N,  111° 40’ 24”W Northwest Territories, Canada | **4** | **4** | Found in the Southern Arctic above treeline, continuous permafrost. Ecozone mean annual temperature: -9.8 C; Ecozone total annual precipitation: 313 mm. |
| *Lake Hazen, NU (LH)* | 81° 49’ 30”N,  71° 19’ 26”W Nunavut, Canada | **17** | **2** | Samples taken from piezometer installed at deepest extent of active-layer (~0.25m). Location was in a subcatchment wetland containing organic-rich soil. Flow direction was through wetland into nearby lake. Organic-rich layer underlain by silt or clay material. |
