# Supplementary material for: Composition Wheels: Visualizing dissolved organic matter using common composition metrics across a variety of Canadian ecozones
Source: PLoS One. 2021 Jul 9;16(7):e0253972. doi: 10.1371/journal.pone.0253972 (PMC8270205; doi:10.1371/journal.pone.0253972)
Supplement: S2 Table — Results from a linear model of dissolved organic matter (DOM) composition (SUVA, slope between 275-295nm, DOC:DON, and humic substances fraction) as predicted by the overall DOM concentration (mg C/L; log transformed). (DOCX) [file pone.0253972.s003.docx]

**S2 Table. DOM Composition versus Concentration Statistics**. Results from a linear model of dissolved organic matter (DOM) composition (SUVA, slope between 275-295nm, DOC:DON, and humic substances fraction) as predicted by the overall DOM concentration (mg C/L; log transformed).

| **Parameter** | **Linear Regression to log(DOM)** | |
| --- | --- | --- |
|  | R^2^ | p-value |
| SUVA | 0.10 | <0.01 |
| S275 | 0.01 | 0.61 |
| DOC:DON | 0.33 | <0.01 |
| HSF | 0.09 | <0.01 |
