# Supplementary material for: Composition Wheels: Visualizing dissolved organic matter using common composition metrics across a variety of Canadian ecozones
Source: PLoS One. 2021 Jul 9;16(7):e0253972. doi: 10.1371/journal.pone.0253972 (PMC8270205; doi:10.1371/journal.pone.0253972)
Supplement: S1 File — Includes DOM concentration (mg C/L), SUVA, slope between 275-295nm, DOC:DON, and humic substances fraction. (DOCX) [file pone.0253972.s004.docx]

Metadata for datasets of Aukes & Schiff Manuscript

| **Title of Dataset** | Data used in 'Composition Wheels: Visualizing dissolved organic matter using common composition metrics across a variety of Canadian Ecozones' |
| --- | --- |
| **URL of Dataset** | TBD |
| **Abstract** | Dissolved organic matter (DOM) is a ubiquitous component of aquatic systems, impacting aquatic health and drinking water quality. These impacts depend on the mixture of organic molecules that comprise DOM. Changing climates are altering both the amount and character of DOM being transported from the terrestrial system into adjacent surface waters, yet DOM composition is not monitored as often as overall concentration. Many DOM characterization methods exist, confounding comparison of DOM composition across different studies. The objective of this research is to determine which parameters in a suite of relatively simple and common DOM characterization techniques explain the most variability in DOM composition from surface and subsurface sites. Further, we create a simple visualization tool to easily compare compositional differences in DOM. A large number of water samples (n=250) was analyzed from six Canadian ecozones for DOM concentration, ultraviolet-visible light absorbance, molecular size, and elemental ratios. Principal component analyses was used to identify quasi-independent DOM compositional parameters that explained the highest variability in the dataset: spectral slope, specific-UV absorbance at 255nm, humic substances fraction, and dissolved organic carbon to dissolved organic nitrogen ratio. A ‘Composition Wheel’ was created by plotting these four parameters as a polygon. Our results find similarities in DOM composition irrespective of site differences in vegetation and climate. Composition Wheels reveal two main shapes that correspond to common compositions of DOM regardless of site: DOM in the subsurface and DOM influenced by photodegradation The Composition Wheel approach uses easily visualized differences in polygon shape to quantify how DOM evolves by natural processes along the aquatic continuum and to track sources and degradation of DOM. |
| **Keywords** | Dissolved organic matter, Canadian ecozones, DOM composition, limnology, freshwater, surface and groundwater, |
| **Dataset Lead Author** | Pieter J. K. Aukes |
| **Position of Data Author** | Graduate Student / Postdoctoral Researcher |
| **Address of Data Authors** | During Data Collection: Department of Earth & Environmental University of Waterloo 200 University Avenue Waterloo, ON N2L 3G1 Canada  Current: Department of Geography and Environmental Studies Wilfrid Laurier University 75 University Avenue West Waterloo, ON N2L 3C5 Canada |
| **E-mail of Data Author** | paukes@uwaterloo.ca |
| **Primary Contact** | Pieter J. K. Aukes |
| **Usage Rights** | Publicly available and free to use |
| **Geographic Region** | Spans various ecoregions across Canada (Northern Arctic, Southern Arctic, Taiga Shield, Boreal Shield, Mixedwood Plains, Atlantic Maritime) |
| **Geographic Coverage** | Lake Hazen, NU – 81° 50’ 50”N 71° 09’ 55”W  Mackenzie River, NT – 69° 29’ 00”N 134° 40’ 00”W  Daring Lake, NT – 64° 31’ 29”N 111° 40’ 24”W  Wekweètì, NT – 64° 11’ 24”N 114° 11’ 10”W  Yellowknife, NT – 62° 27’ 14”N 114° 22’ 18”W  Turkey Lakes Watershed, ON – 47° 2’ 54”N 84° 24’ 25”W  IISD Experimental Lakes Area, ON – 49° 39’ 40”N 93° 43’ 48”W  Nottawasaga River Watershed, ON – 44° 7’ 26”N 79° 49’ 12”W  Grand River Watershed, ON – 43° 30’ 41”N 80° 29’ 43”W  Black Brook Watershed, NB – 47° 6’ 11”N 67° 45’ 40”W |
| **Temporal Coverage** | 2013-05-01 to 2016-10-01 |
| **General Study Design** | Collection of field samples (surface and groundwaters) from a variety of ecoregions (representing differences in vegetation, mean annual temperature, mean annual precipitation, geology). Different measures of DOM composition were applied to all samples, and statistics was used to determine which suite of measures best represents DOM composition across a gradient of environments. We then used these measures to develop a visualization tool to easily compare differences in DOM composition (‘Composition Wheel’). |
| **Methods** | **Field Processing**  Water collected using a syringe and filtered in-field to 0.45μm (Whatman GD/X) into pre-rinsed, acid-washed 40 mL glass vials. Samples were kept cool (<4C) and in the dark until analyses.  **Laboratory Analyses – DOM Concentration & Composition**  Dissolved organic carbon and total nitrogen concentrations were measured using a Shimadzu Total Organic Carbon (TOC-L) Combustion Analyzer with TNM-1 module. Dissolved organic nitrogen (DON) was calculated as the difference between total dissolved nitrogen concentration and the sum of inorganic nitrogen species (nitrate, nitrite, and ammonium). Inorganic nitrogen species were measured using SmartChem 200 Automated Chemistry Analyzer (Unity Scientific, MA United States). The DOC:DON ratio was calculated using molar concentrations of DOC (MC) and DON (MN).  Absorbance was measured using a Cary 100 UV-VIS Spectrophotometer (Agilent, CA United States) at 5 nm increments from 200 to 800 nm. Deionized water was used to zero the instrument and run intermittently during analyses to correct for baseline drift. The Naperian absorption coefficient (a; m-1) was calculated using:  a_λ=(ln(10)×A_λ)/L  where A is the baseline-corrected absorbance at wavelength λ and L is the cell length (m). A suite of absorbance characteristics were then calculated.  Molecular-size based fractions of DOM were determined using a size exclusion chromatography technique (Liquid Chromatography – Organic Carbon Detection, LC-OCD) at the University of Waterloo. Detailed instrument setup and analysis is described elsewhere. Briefly, the sample was injected through a size-exclusion column (SEC; Toyopearl HW-50S, Tosoh Bioscience) that separated DOM based on hydrodynamic radii into five hydrophilic fractions (from largest to smallest): biopolymers (BP; polysaccharides or proteins), humic substances fraction (HSF; humic and fulvic acid fraction), building blocks (BB; lower weight humic substances), low molecular weight neutrals (LMWN; aldehydes, small organic materials), and LMW-acids (LMWA; saturated mono-protic acids). A portion of the sample by-passes the SEC for determination of the overall DOC concentration, here referred to as DOM concentration in mg C/L. A number average molecular weight was derived only for the HSF based on elution time. Duplicates run at six concentrations yield a precision for the LC-OCD of <0.1 mg C/L for all fractions. Concentrations of each fraction were calculated using specialized software (ChromCALC, DOC-Labor, Germany) that integrated chromatograms from the LC-OCD.  **Statistics & Composition Wheel Design**  Samples from sites with multiple sampling events were averaged to create a single value per site. Data were analysed using unconstrained ordination analysis via principal components analysis (PCA) on a subset of samples that contained all composition measures (subset n=130). Data were scaled before PCA and analysed using R Statistical Software.  The Composition Wheel (CW) is a polygon drawn from axes of various composition measures that are independent of DOM concentration in order to focus solely on differences in DOM composition. Composition Wheel parameters were chosen based on the highest contribution of variables explaining the first two principal component axes. Further, independent measures of DOM composition were preferentially chosen to minimize overlap in information between similar techniques. Each CW axis corresponds to a specific parameter. For each axis, the individual value for each sample is normalized as a value between the maximum and minimum encountered for that parameter within the dataset. Code used to create the DOM CW can be found at used at <https://github.com/paukes/DOM-Comp-Wheel>. |
| **Quality Control** | Samples were taken in duplicate. A series of standards was run with all samples to correct for concentration. For LC-OCD analyses, DOM concentration standards and IHSS-HA and IHSS-FA were run on the same run as the samples. For UV-VIS absorbance, deionized water was run intermittently to correct for baseline drift. For all, certain samples were run twice to assess the precision of the machine. |
| **Included Files** | *‘Aukes_Schiff_DOMcompare.csv’* – comparison of specific UV-absorbance at 255nm (SUVA), spectral slope, humic substances fraction, and DOC:DON across different Canadian ecozones.  Data  *‘Aukes_Schiff_PCA.csv’* – Subset of 'Aukes_Schiff_DOMcompare.csv' for PCA analysis. These samples include values for all DOM composition measures (size-exclusion chromatography, DOC:DON, UV-VIS absorbance parameters).  *‘spencer et al 2012.csv’* – Data used to compare to our results, from Spencer RGM, Butler KD, Aiken GR. Dissolved organic carbon and chromophoric dissolved organic matter properties of rivers in the USA. J Geophys Res Biogeosciences. 2012;117. doi:10.1029/2011JG001928  *‘jaffe et al 2008.csv’* – Data used to compare to our results, from Jaffé R, McKnight D, Maie N, Cory RM, McDowell WH, Campbell JL. Spatial and temporal variations in DOM composition in ecosystems: The importance of long-term monitoring of optical properties. J Geophys Res. 2008;113: 1–15. doi:10.1029/2008JG000683 |

Description of variables in the datasets

| **File** | **Column Name** | **Definition** | **Units** |
| --- | --- | --- | --- |
| Aukes_Schiff_DOMcompare.csv | Env | Surface or groundwater |  |
| Aukes_Schiff_DOMcompare.csv | Location | Geographic location where sampled |  |
| Aukes_Schiff_DOMcompare.csv | Hydro | Water body type |  |
| Aukes_Schiff_DOMcompare.csv | Area | Specific area of collection within location |  |
| Aukes_Schiff_DOMcompare.csv | Site | Unique sample name |  |
| Aukes_Schiff_DOMcompare.csv | DOC_mg.L | Dissolved organic carbon concentration | mg C/L |
| Aukes_Schiff_DOMcompare.csv | SUVA | Specific ultraviolet absorbance at 255 nm | L/(mg m) |
| Aukes_Schiff_DOMcompare.csv | S275.295 | Spectral slope between 275 and 295nm | m^-1^ |
| Aukes_Schiff_DOMcompare.csv | DOC:DON | Molar ratio of dissolved organic carbon to dissolved organic nitrogen |  |
| Aukes_Schiff_DOMcompare.csv | HS. | Proportion of humic substances fraction, determined via LC-OCD |  |
| Aukes_Schiff_DOMcompare.csv | BP. | Proportion of biopolymer fraction, determined via LC-OCD |  |
| Aukes_Schiff_DOMcompare.csv | LMWN. | Proportion of low molecular weight fraction, determined via LC-OCD |  |
| Aukes_Schiff_PCA.csv | Year | Year of sample collection |  |
| Aukes_Schiff_PCA.csv | Date | Date of sample collection |  |
| Aukes_Schiff_PCA.csv | Month | Month of sample collection |  |
| Aukes_Schiff_PCA.csv | Location | Geographic location where sampled |  |
| Aukes_Schiff_PCA.csv | Env | Surface or groundwater |  |
| Aukes_Schiff_PCA.csv | Hydro | Water body type |  |
| Aukes_Schiff_PCA.csv | Area | Specific area of collection within location |  |
| Aukes_Schiff_PCA.csv | SampleID | Unique sample name |  |
| Aukes_Schiff_PCA.csv | DOC_mg.L | Dissolved organic carbon concentration | mg C/L |
| Aukes_Schiff_PCA.csv | DOC:DON | Molar ratio of dissolved organic carbon to dissolved organic nitrogen |  |
| Aukes_Schiff_PCA.csv | Hphi | Concentration of all eluted components, determined via LC-OCD | mg C/L |
| Aukes_Schiff_PCA.csv | Hphi. | Proportion of all eluted components (compared to overall DOC), determined via LC-OCD |  |
| Aukes_Schiff_PCA.csv | BP | Concentration of biopolymer fraction, determined via LC-OCD | mg C/L |
| Aukes_Schiff_PCA.csv | BP. | Proportion of the biopolymer fraction, determined via LC-OCD |  |
| Aukes_Schiff_PCA.csv | HS | Concentration of humic substances fraction, determined via LC-OCD | mg C/L |
| Aukes_Schiff_PCA.csv | HS. | Proportion of the humic substances fraction, determined via LC-OCD |  |
| Aukes_Schiff_PCA.csv | HS.SUVA | Specific UV-absorbance at 255nm for the humic substances fraction only | L/(mg m) |
| Aukes_Schiff_PCA.csv | HS.MW | Nominal molecular weight for the humic substances fraction | g/mol |
| Aukes_Schiff_PCA.csv | BB | Concentration of building blocks fraction, determined via LC-OCD | mg C/L |
| Aukes_Schiff_PCA.csv | BB. | Proportion of the building blocks fraction, determined via LC-OCD |  |
| Aukes_Schiff_PCA.csv | LMWN | Concentration of low molecular weight neutral fraction, determined via LC-OCD | mg C/L |
| Aukes_Schiff_PCA.csv | LMWN. | Proportion of the low molecular weight neutral fraction, determined via LC-OCD |  |
| Aukes_Schiff_PCA.csv | LMWA | Concentration of low molecular weight acids fraction, determined via LC-OCD | mg C/L |
| Aukes_Schiff_PCA.csv | LMWA. | Proportion of the low molecular weight acids fraction, determined via LC-OCD |  |
| Aukes_Schiff_PCA.csv | SUVA | Specific ultraviolet absorbance at 255 nm | L/(mg m) |
| Aukes_Schiff_PCA.csv | E2:E3 | Absorbance at 255nm divided by absorbance at 365nm |  |
| Aukes_Schiff_PCA.csv | E4:E6 | Absorbance at 465nm divided by absorbance at 665nm |  |
| Aukes_Schiff_PCA.csv | SAC350 | The absorbance at 350 nm normalized to DOM concentration (specific absorption at 350 nm) | L/(mg m) |
| Aukes_Schiff_PCA.csv | TrueColour | Measure of True Colour | True Colour Units |
| Aukes_Schiff_PCA.csv | S275-295 | Spectral slope between 275 and 295nm | m^-1^ |
| Aukes_Schiff_PCA.csv | S350-400 | Spectral slope between 350 and 400nm | m^-1^ |
| Aukes_Schiff_PCA.csv | Sr | Slope ratio (S275-295 divided by S350-400) |  |
| Aukes_Schiff_PCA.csv | Abs420 | The absorbance at 420 m, | m^-1^ |
| Aukes_Schiff_PCA.csv | SAC420 | The absorbance at 420 nm normalized to DOM concentration (specific absorption at 420 nm) | L/(mg m) |
| Jaffe et al 2008.csv | DOC | Compiled data on concentration of dissolved organic matter | mg C/L |
| Jaffe et al 2008.csv | SUVA | Compiled data on specific ultraviolet absorbance at 255 nm | L/(mg m) |
| Spencer et al 2012.csv | DOC |  |  |
| Spencer et al 2012.csv | SUVA | Compiled data on specific ultraviolet absorbance at 255 nm | L/(mg m) |
| Spencer et al 2012.csv | s275.295 | Compiled data on spectral slope between 275 and 295nm | nm^-1^ |
